# Prevalence, risk factors and molecular evaluation of hepatitis E virus infection among pregnant women resident in the northern shores of Persian Gulf, Iran

**DOI:** 10.1371/journal.pone.0191090

**Published:** 2018-01-12

**Authors:** Fatemeh Farshadpour, Reza Taherkhani, Mohamad Reza Ravanbod, Seyed Sajjad Eghbali, Sakineh Taherkhani, Easa Mahdavi

**Affiliations:** 1 The Persian Gulf Tropical Medicine Research Center, Bushehr University of Medical Sciences, Bushehr, Iran; 2 Department of Internal Medicine, School of Medicine, Bushehr University of Medical Sciences, Bushehr, Iran; 3 Reproductive Health and Midwifery Department, School of Nursing and Midwifery, Shahroud University of Medical Sciences, Shahroud, Iran; 4 Student Research Committee, Bushehr University of Medical Sciences, Bushehr, Iran; University of Cincinnati College of Medicine, UNITED STATES

## Abstract

**Background:**

Although Iran is reported to be an endemic country for hepatitis E virus (HEV), data on the prevalence of HEV infection among pregnant women are scarce and the epidemiology of HEV is unknown in most parts of the country. Therefore, this study was conducted to elucidate the prevalence, risk factors and genotypic pattern of HEV infection among pregnant women resident in the northern shores of Persian Gulf. This is the first report on the epidemiology of HEV infection among pregnant women in this territory.

**Methods:**

From October 2016 to May 2017, 1331 pregnant women participated in this study. The mean age ± SD of participants was 27.93±5.7 years with a range of 14–45 years. Serum samples of pregnant women were screened for the presence of anti-HEV total antibodies, anti-HEV IgG and anti-HEV IgM using commercially available ELISA kits (DIA.PRO, Milan, Italy). All anti-HEV IgG and anti-HEV IgM positive samples were tested for HEV RNA using two independent reverse transcriptase polymerase chain reaction (RT-PCR) assays, targeting ORF2 and ORF3 of HEV genome. In addition, 92 anti-HEV seronegative samples as well as 50 pooled seronegative samples were evaluated by two independent RT-PCR assays for validation of results.

**Results:**

Of the 1331 pregnant women, 84 women (6.3%, 95% CI: 5.1%-7.7%) were positive for anti-HEV antibodies, of which 83 women had anti-HEV IgG, and 11 women (0.83%, 95% CI: 0.47%-1.47%) had anti-HEV IgM. The highest rate of HEV seroprevalence was observed among Afghan immigrants (68.0%), uneducated pregnant women (46.51%) and those residents in Bushehr city (8.75%). All anti-HEV IgG and/or IgM positive samples were found to be negative for HEV RNA. In addition, all of the evaluated anti-HEV seronegative samples were negative for HEV RNA. HEV seropositivity among pregnant women was statistically associated with age, ethnicity, place of residence, number of pregnancies, and level of education. So that, low education levels, Afghan, residence in Bushehr city, age group >34 years, and more parities were risk factors for exposure to HEV. In contrast, HEV seropositivity was not associated with stage of gestation, history of abortion, and time of sampling.

**Conclusion:**

The northern shores of Persian Gulf in Iran, with HEV seroprevalence of 6.3%, can be classified as an endemic geographical region for hepatitis E, and residents of Bushehr city, Afghan immigrants and uneducated women are the main at-risk populations in this territory.

## Introduction

Hepatitis E virus (HEV), a member of the family *Hepeviridae*, is characterized by an acute self-limited or asymptomatic course in the host. HEV is known to cause large outbreaks. Complications are usually self-limiting, and the treatment is mainly supportive [1, 2]. Although Hepatitis E outbreaks have mostly been described in developing countries so far, the virus is already known to circulate in developed countries as silent infection [2, 3]. HEV is usually acquired through consumption of contaminated food or water. In addition to faecal-oral route, organ transplantation, blood transfusion, hemodialysis and sexual intercourse are some other possible ways of HEV transmission [4, 5].

Clinical presentations of HEV infection are minimal in the general population but can lead to life-threatening conditions in immunocompromised patients, pregnant women, organ transplant recipient patients and those with underlying liver problems [4]. The maternal mortality rate of HEV infection during pregnancy can reach to 20–25% accompanying with prenatal or neonatal complications such as stillbirth, miscarriage, death after birth, premature birth, and jaundice [2]. Such complications are well known trigger of HEV infection during pregnancy, however, little is known about the underlying pathomechanisms. Progression to such severe complications during pregnancy seems to be the consequence of direct or indirect cytopathic effects of HEV through immune-mediated pathogenesis. The pregnancy-related changes in immunological and hormonal responses have also a profound effect on the pathogenesis of HEV. Further studies are needed to shed more light on the underlying mechanisms [1, 6].

The importance of HEV infection during pregnancy as a health dilemma is well known, but most of the time this importance is neglected maybe due to anomalous observations on hepatitis E complications among pregnant women in different parts of the world [7]. To reach a general consensus on this infection during pregnancy, HEV surveillance in all parts of the world is needed in order to provide appropriate public health recommendations. Although Iran is reported to be an endemic country for HEV, data on the prevalence of HEV infection among pregnant women in Iran are scarce and the epidemiology of HEV is unknown in most parts of the country [8, 9]. Therefore, this study was conducted to elucidate the prevalence, risk factors and genotypic pattern of HEV infection among pregnant women in South of Iran, the northern shores of Persian Gulf. This is the first report on the epidemiology of HEV infection among pregnant women in this territory.

## Material and methods

### Study setting and population

This descriptive cross-sectional study was funded by Bushehr University of Medical Sciences through research grant number 3253. From October 2016 to May 2017, the population-based sample of pregnant women from Bushehr province was collected randomly using the multistage cluster sampling method. Bushehr is a large province in the northern shores of Persian Gulf, which consists of 10 cities with different ethnicities including Fars, Arab and Turk. In addition, a huge number of Afghan immigrants are resident in this region.

In the first stage, four of the most populated cities of this territory, including Bushehr, Borazjan, Ahram and Jam were selected randomly. In the next stage, two public health centers were selected randomly from each city. In the mentioned time period, consecutive pregnant women attending these public health centers for routine gynecological screening were enrolled in this study. A questionnaire containing demographic information of age, place of residence, ethnicity, stage of gestation, number of pregnancy, history of abortion, level of education and time of sampling was completed for each participant. In addition, all pregnant women were requested to give a written informed consent to use their leftover serum samples for HEV detection and use of test results for analysis. The ethical committee of Bushehr University of Medical Sciences approved this study (reference no. IR.BPUMS.Rec.1395.47). The records of all participants were analyzed in an anonymous way with respect to the results of serological screening for HEV infection.

### Laboratory diagnosis

Serum samples of pregnant women who gave written informed consent were screened for the presence of anti-HEV total antibodies, anti-HEV IgG and anti-HEV IgM using commercially available ELISA kits (HEV Ab ELISA kit, HEV IgG ELISA kit and HEV IgM ELISA kit, DIA.PRO, Milan, Italy). The specificity of the HEV Ab, HEV IgM and HEV IgG DIA.PRO ELISA kits was > 99.5%, > 95% and > 99.5%, respectively, and the sensitivity of these kits was 100%. In addition, the DIA.PRO ELISA had high concordance compared with the in-house ELISA [10]. All anti-HEV IgG and anti-HEV IgM positive samples were tested for HEV RNA using two independent reverse transcriptase polymerase chain reaction (RT-PCR) assays. In addition, 92 anti-HEV seronegative samples as well as 50 pooled seronegative samples were evaluated by two independent RT-PCR assays for validation of results. Two hundred seronegative samples were combined into pools of 4 using 50μl of each sample. First, the samples were subjected to an in house nested RT-PCR, targeting ORF2/ORF3 of HEV genome. Briefly, HEV RNA was extracted from serum samples using High Pure Viral Nucleic Acid kit (Roche, Mannheim, Germany) and was immediately reverse-transcribed into cDNA using SuperScript III cDNA synthesis kit (Invitrogen, Carlsbad, CA, USA) according to the manufacturer’s instructions. Then, cDNA was amplified by nested RT-PCR using outer primers [forward primer (HE361): GCRGTGGTTTCTGGGGTGAC; reverse primer (HE364): CTGGGMYTGGTCDCGCCAAG] and inner primers [forward primer (HE366): GGGYTGATTCTCAGCCCTTCGC; reverse primer (HE363): GMYTGGTCDCGCCAAGHGGA].

The samples were further tested using another nested RT-PCR. The following sets of nested primers were used to amplify 731 nucleotides and 348 nucleotides of the ORF2 region, respectively: outer primers [forward primer (3156N): AATTATGCYCAGTAYCGRGTTG; reverse primer (3157N): CCCTTRTCYTGCTGMGCATTCTC] and inner primers [forward primer (3158N): GTWATGCTYTGCATWCATGGCT; reverse primer (3159N): AGCCGACGAAATCAATTCTGTC]. The sequences of primers, regions in genome and PCR conditions for detection of HEV RNA are summarized in Table 1. The amplified 348 bp length fragment was then submitted for sequencing to determine HEV genotypes. Precise procedures and protocols were taken into consideration in order to avoid potential carry-over contaminations in the cDNA synthesis and PCR reactions [11]. In addition, negative and positive controls were included in each round of PCR to validate results.

**Table 1:** Sequences of primers for detection of hepatitis E virus.

Virus	Primers Name	Sequences of Primers 5′→3′	Gene	Region in Genome	Annealing temperature	Size	References
HEV	HE361	GCRGTGGTTTCTGGGGTGAC	ORF2/ORF3	5257–5276	56 C	164 bp	[12]
	HE364	CTGGGMYTGGTCDCGCCAAG		5420–5401			
	HE366	GGGYTGATTCTCAGCCCTTCGC		5278–5299	56 C	139 bp	
	HE363	GMYTGGTCDCGCCAAGHGGA		5416–5397			
HEV	3156N	AATTATGCYCAGTAYCGRGTTG	ORF2	5685–5706	55 C	731 bp	[13]
	3157N	CCCTTRTCYTGCTGMGCATTCTC		6415–6393			
	3158N	GTWATGCTYTGCATWCATGGCT		5970–5991	55 C	348 bp	
	3159N	AGCCGACGAAATCAATTCTGTC		6317–6296			

### Statistical analysis

SPSS 17 package program (SPSS Inc., Chicago, IL, USA) was used to perform all statistical analyses, and *P* values of less than 0.05 were defined statistically significant. Data were presented as frequencies, percentage and mean ± standard deviation (SD) following analysis by descriptive statistics. Quantitative data were compared between HEV seropositive and HEV seronegative pregnant women by Student's t-test. Chi-square test or Fisher’s exact test was used to compare and analyze categorical data. To evaluate the effect of variables on HEV seropositivity and to identify the risk factors of HEV infection in pregnant women, logistic regression analysis was used, and odds ratio (OR) with 95% confidence intervals (CI) was calculated.

## Result

During the study period, 1331 pregnant women participated in this study. The mean age ± SD of participants was 27.93±5.7 years with a range of 14–45 years. The pregnant women were classified into five age groups: <20, 20–24, 25–29, 30–34 and over 34 years. The majority of pregnant women were in the age group 25–29 years (34.9%), third trimester of pregnancy (54.0%), as well as Fars (83.3%) and educated. Of the 1331 pregnant women, 84 women (6.3%, 95% CI: 5.1%-7.7%) were positive for anti-HEV antibodies, of which 83 women had anti-HEV IgG, and 11 women (0.83%, 95% CI: 0.47%-1.47%) had anti-HEV IgM. All anti-HEV IgM seropositive samples were positive for anti-HEV IgG except one sample, which was positive for anti-HEV IgM and negative for anti-HEV IgG. All anti-HEV IgG and/or IgM positive samples were found to be negative for the presence of HEV RNA. In addition, all of the evaluated anti-HEV seronegative samples were negative for HEV RNA. Regarding the pregnancy outcome, all pregnant women had successful childbirth except eight seronegative pregnant women. These eight women had spontaneous abortion. Nevertheless, these women were negative for HEV RNA despite experiencing abortion.

When we evaluated the seroprevalence of HEV in the age groups, the highest rate of anti-HEV seroprevalence was found in the age group >34 years (11.76%) followed by the age group 20–24 years (8.74%), while pregnant women in the age groups 30–34 years (3.45%) and <20 years (3.49%) showed the lowest anti-HEV seropositivity. The seroprevalence of anti-HEV IgM decreased with age from 2.8% in women aged 20–24 years to 0.49% in women over 34 years old. Overall, anti-HEV seropositive pregnant women showed higher mean age (28.80±7.1) compared to HEV seronegative women (27.87±5.6), but this difference was not statistically significant (*P* = 0.15). While anti-HEV IgM seropositive pregnant women had significantly lower mean age (22.45±5.26) compared to anti-HEV IgM seronegative women (27.98±5.7) (*P* = 0.001).

Regarding the place of residence, the highest prevalence rates of anti-HEV IgM and anti-HEV total antibodies were observed among pregnant women resident in Bushehr city (1.46% and 8.75%, respectively), while Jam city had the lowest prevalence rates for anti-HEV IgM and anti-HEV total antibodies among pregnant women in this region (0.0% and 1.8%, respectively). HEV seroprevalence showed a decreasing prevalence with education level, so that pregnant women with a higher education level had lower seroprevalence of anti-HEV total antibodies (1.17%) and anti-HEV IgM (0.0%) when compared to uneducated women (46.51% and 11.63%, respectively). According to ethnicity, Afghan women had the highest seropositivity (68.0% for anti-HEV total antibodies and 16.0% for anti-HEV IgM antibody); while Arab women showed the lowest seroprevalence rates (3.16% for anti-HEV total antibodies and 0.0% for anti-HEV IgM). Overall, the highest rate of HEV seroprevalence was observed among Afghan and uneducated pregnant women. In addition, the seroprevalence of anti-HEV antibodies increased with the number of pregnancy, so that women with more than three pregnancies showed the highest HEV seropositivity (12.7%).

The majority of HEV seropositive pregnant women were in their second trimester of pregnancy and had history of abortion, while, in the logistic regression analysis, none of these parameters were significantly associated with HEV seropositivity. Anti-HEV total antibodies were more likely to be positive in those samples that collected during November (9.62%), May (9.62%) and January (9.57%). As for anti-HEV IgM, those samples collected during January showed the highest seropositivity (2.6%). Nevertheless, no significant association was identified between HEV seropositivity and time of sampling.

HEV seropositivity among pregnant women was statistically associated with age, ethnicity, place of residence, number of pregnancies and level of education. So that, low education levels (OR: 8.48; 95% CI: 4.33–16.58; *P* < 0.001 for uneducated women and OR: 3.89; 95% CI: 2.18–6.97; *P* < 0.001 for under diploma women), Afghan (OR: 65.03; 95% CI: 22.3–189.7; *P* < 0.001), residence in Bushehr city (OR: 2.09; 95% CI: 1.23–3.55; *P* = 0.006), age group >34 years (OR: 3.73; 95% CI: 1.74–7.99; *P* = 0.001) and more than three pregnancies (OR: 2.54; 95% CI: 1.48–4.36; *P* = 0.001) were significant predictive variables for HEV seropositivity in pregnant women. On the other hand, illiteracy was the only risk factor associated with anti-HEV IgM seropositivity among pregnant women (OR: 16.12; 95% CI: 4.16–62.52; *P* < 0.001). The prevalence rates of anti-HEV total antibodies and anti-HEV IgM among pregnant women grouped according to socio-demographic characteristics are shown in Tables 2 and 3, respectively.

**Table 2:** Prevalence of anti-HEV total antibodies (IgG+IgM) among pregnant women according to socio-demographic characteristics.

	No. of all participants (%): 1331 (100%)	No. of HEV total Ab negative subjects (%): 1247 (93.7%)	No. of HEV total Ab positive subjects (%): 84 (6.3%)	Adjusted OR (95% CI)	*P-Value*
**Age groups (years)**					
<20	86 (6.5%)	83 (96.51%)	3 (3.49%)	1.0	
20–24	286 (21.5%)	261 (91.26%)	25 (8.74%)	2.65 (0.78–9.0)	0.12
25–29	465 (34.9%)	443 (95.27%)	22 (4.73%)	0.52 (0.29–0.94)	0.03
30–34	290 (21.8%)	280 (95.55%)	10 (3.45%)	0.72 (0.34–1.54)	0.4
>34	204 (15.3%)	180 (88.24%)	24 (11.76%)	3.73 (1.74–7.99)	0.001
**Place of residence (city)**					
Borazjan	456 (34.3%)	436 (95.61%)	20 (4.39%)	1.0	
Bushehr	617 (46.4%)	563 (91.25%)	54 (8.75%)	2.09 (1.23–3.55)	0.006
Ahram	147 (11.0%)	139 (94.56%)	8 (5.44%)	0.60 (0.28–1.29)	0.19
Jam	111 (8.3%)	109 (98.2%)	2 (1.8%)	0.32 (0.07–1.53)	0.15
**Ethnicity**					
Fars	1109 (83.3%)	1065 (96.03%)	44 (3.97%)	1.0	
Arab	158 (11.9%)	153 (96.84%)	5 (3.16%)	0.79 (0.31–2.03)	0.63
Afghan	50 (3.8%)	16 (32.0%)	34 (68.0%)	65.03 (22.3–189.7)	0.000
Turk	14 (1.1%)	13 (92.86%)	1 (7.14%)	0.04 (0.004–0.3)	0.002
**Stage of gestation**					
First trimester	352 (26.4%)	332 (94.32%)	20 (5.68%)	1.0	
Second trimester	260 (19.5%)	237 (91.15%)	23 (8.85%)	1.61 (0.86–3.0)	0.13
Third trimester	719 (54.0%)	678 (94.3%)	41 (5.7%)	0.62 (0.37–1.06)	0.08
**Number of pregnancies**					
One pregnancy	412 (31.0%)	391 (94.9%)	21 (5.1%)	1.0	
Two and three pregnancies	738 (55.4%)	698 (94.6%)	40 (5. 4%)	1.1 (0.62–1.84)	0.81
More than three pregnancies	181 (13.6%)	158 (87.3%)	23 (12.7%)	2.54 (1.48–4.36)	0.001
**History of abortion**					
No	633 (47.6%)	605 (95.58%)	28 (4.42%)	1.0	
Yes	162 (12.2%)	153 (94.44%)	9 (5.56%)	1.27 (0.59–2.75)	0.54
Unknown	536 (40.3%)	489 (91.23%)	47 (8.77%)	1.63 (0.78–3.41)	0.19
**Education**					
Upper diploma	171 (12.8%)	169 (98.83%)	2 (1.17%)	1.0	
Diploma	623 (46.8%)	607 (97.43%)	16 (2.57%)	2.23 (0.51–9.78)	0.29
Under diploma	494 (37.1%)	448 (90.69%)	46 (9.31%)	3.89 (2.18–6.97)	0.000
Uneducated	43 (3.2%)	23 (53.49%)	20 (46.51%)	8.48 (4.33–16.58)	0.000
**Month**					
Oct	113 (8.5%)	109 (96.46%)	4 (3.54%)	1.0	
Nov	208 (15.6%)	188 (90.38%)	20 (9.62%)	0.34 (0.12–1.04)	0.06
Dec	249 (18.7%)	241 (96.79%)	8 (3.21%)	1.11 (0.37–3.75)	0.87
Jan	115 (8.6%)	104 (90.43%)	11 (9.57%)	0.35 (0.11–1.12)	0.08
Feb	207 (15.6%)	197 (95.17%)	10 (4.83%)	0.72 (0.22–2.36)	0.59
Mar	229 (17.2%)	214 (93.45%)	15 (6.55%)	0.52 (0.17–1.62)	0.26
Apr	158 (11.9%)	147 (93.04%)	11 (6.96%)	0.49 (0.15–1.58)	0.23
May	52 (3.9%)	47 (90.38%)	5 (9.62%)	0.34 (0.09–1.34)	0.12

**Table 3:** Prevalence of anti-HEV IgM among pregnant women according to socio-demographic characteristics.

	No. of all participants (%): 1331 (100%)	No. of HEV IgM negative subjects (%): 1320 (99.17%)	No. of HEV IgM positive subjects (%): 11 (0.83%)	Adjusted OR (95% CI)	*P*-Value
**Age groups (years)**					
<20	86 (6.5%)	84 (97.67%)	2 (2.33%)	1.0	
20–24	286 (21.5%)	278 (97.2%)	8 (2.8%)	0.83 (0.17–3.97)	0.813
25–29	465 (34.9%)	465 (100.0%)	0 (0.0%)	0.00	0.993
30–34	290 (21.8%)	290 (100.0%)	0 (0.0%)	0.00	0.994
>34	204 (15.3%)	203 (99.51%)	1 (0.49%)	4.83 (0.43–54.02)	0.201
**Place of residence (city)**					
Borazjan	456 (34.3%)	455 (99.78%)	1 (0.22%)	1.0	
Bushehr	617 (46.4%)	608 (98.54%)	9 (1.46%)	6.73 (0.85–53.35)	0.07
Ahram	147 (11.0%)	146 (99.32%)	1 (0.68%)	0.46 (0.06–3.68)	0.47
Jam	111 (8.3%)	111 (100.0%)	0 (0.0%)	0.00	0.99
**Ethnicity**					
Fars	1109 (83.3%)	1106 (99.73%)	3 (0.27%)	1.0	
Arab	158 (11.9%)	158 (100.0%)	0 (0.0%)	0.00	0.99
Afghan	50 (3.8%)	42 (84.0%)	8 (16.0%)	0.02 (0.01–0.06)	0.000
Turk	14 (1.1%)	14 (100.0%)	0 (0.0%)	0.00	0.99
**Stage of gestation**					
First trimester	352 (26.4%)	349 (99.15%)	3 (0.85%)	1.0	
Second trimester	260 (19.5%)	256 (98.46%)	4 (1.54%)	1.82 (0.4–8.19)	0.44
Third trimester	719 (54.0%)	715 (99.44%)	4 (0.56%)	0.36 (0.09–1.44)	0.15
**Number of pregnancies**					
One pregnancy	412 (31.0%)	408 (99.03%)	4 (0.97%)		
Two and three pregnancies	738 (55.4%)	731 (99.05%)	7 (0.95%)	0.98 (0.28–3.36)	0.97
More than three pregnancies	181 (13.6%)	181 (100.0%)	0 (0.0%)	0.00	0.99
**History of abortion**					
No	633 (47.6%)	629 (99.37%)	4 (0.63%)		
Yes	162 (12.2%)	161 (99.38%)	1 (0.62%)	0.98 (0.11–8.8)	0.98
Unknown	536 (40.3%)	530 (98.88%)	6 (1.12%)	1.82 (0.22–15.25)	0.58
**Level of education**					
Upper diploma	171 (12.8%)	171 (100.0%)	0 (0.0%)	1.0	
Diploma	623 (46.8%)	621 (99.68%)	2 (0.32%)	0.00	0.99
Under diploma	494 (37.1%)	490 (99.19%)	4 (0.81%)	2.53 (0.46–13.9)	0.28
Uneducated	43 (3.2%)	38 (88.37%)	5 (11.63%)	16.12 (4.16–62.52)	0.00
**Time of sampling (month)**					
Oct	113 (8.5%)	112 (99.11%)	1 (0.89%)	1.0	
Nov	208 (15.6%)	208 (100.0%)	0 (0.0%)	0.00	0.99
Dec	249 (18.7%)	248 (99.6%)	1 (0.4%)	2.21 (0.14–35.72)	0.57
Jan	115 (8.6%)	112 (97.4%)	3 (2.6%)	0.33 (0.03–3.25)	0.35
Feb	207 (15.6%)	205 (99.03%)	2 (0.97%)	0.92 (0.08–10.21)	0.94
Mar	229 (17.2%)	229 (100.0%)	0 (0.0%)	0.00	0.99
Apr	158 (11.9%)	155 (98.1%)	3 (1.9%)	0.46 (0.05–4.49)	0.51
May	52 (3.9%)	51 (98.08%)	1 (1.92%)	0.46 (0.03–7.43)	0.58

## Discussion

HEV is known to cause acute viral hepatitis, but complications are usually self-limiting. HEV is also known for its ability to cause severe life threatening complications in pregnant women and their offspring [7]. Despite its importance during pregnancy, there are limited data on the prevalence and risk factors of HEV infection among pregnant women in Iran [9]. In addition, no data are available so far on HEV prevalence among pregnant women resident in the northern shores of Persian Gulf. Therefore, we evaluated the pregnant women resident in this region in order to determine the prevalence of HEV infection, the possible risk factors associated with incidence of HEV infection and to clarify the epidemiology of HEV infection among pregnant women in this territory.

HEV seroprevalence of 6.3% reported in the present study is higher than those reported among pregnant women in different parts of Iran, 3.6% in Urmia [14] and 5.26% in Ahvaz [15], but lower than those reported in Hamadan (7.4%) [16] and Gorgan (7.4%) [17]. Overall, the prevalence of HEV among pregnant women varies from 3.6% to 7.4% in different regions of Iran. These results demonstrate that the prevalence of HEV infection is variable even within a country and dependent upon level of public health and hygiene of each region [9]. The reported seroprevalence in this study is also higher than HEV seroprevalence among pregnant women in Spain (3.6%) [18], and Mexico (5.7%) [19] but lower than those of Nile Delta (84.3%) [20], Sudan (61.2%) [21], Ethiopia (31.6%) [22], Ghana (28.66%) [23], the United Arab Emirates (20.0%) [24], Benin (16.19%) [25], Gabon (14.1%) [26], Turkey (12.6%) [27], China (11.1%) [28], and France (7.74%) [29].

The seroprevalence of 0.83% for anti-HEV IgM observed in this study is in range of previous reports from different parts of Iran, 0.23% among pregnant women in Ahvaz [15], 0.9% among general population in Shiraz [30], and 1.4% among adults in Ahvaz [31]. This seroprevalence is lower than that reported in Benin (1.44%) [25] but higher than those of Spain (0.67%) [18], China (0.6%) [28], Ethiopia (0.5%) [22], and Turkey (0.0%) [27].

These variations in the prevalence of HEV infection reflect differences in the prevalence of HEV infection in the general population, routes of transmission, risk factors, level of awareness, preventive strategies, socioeconomic status and level of sanitation in different parts of the world [8, 32]. In addition, differences in the time of sampling, number of participants or sample size, sociodemographic characteristics of study population, study period, and specificity and sensitivity of diagnostic assays can explain these variations [8, 31].

The anti-HEV prevalence of 6.3% and anti-HEV IgM prevalence of 0.83% reported in this study are in the range of HEV seroprevalence in the general population of Iran, 1.1% to 14.2% for anti-HEV IgG and anti-HEV total antibodies and 0.0% to 0.9% for anti-HEV IgM [8]. These coincidences considerably indicate that the prevalence of HEV infection in a given population can be influenced by the burden of HEV infection in the general population.

In Iran, as the other endemic country, HEV is predominantly transmitted through contamination of water supplies [8]. This mode of transmission effectively spreads HEV in the society and is responsible for occurrence of epidemics or outbreaks of hepatitis E. While in non-endemic countries, HEV is mainly transmitted through contamination of food supplies and mostly appears as sporadic infection [1, 5, 8]. However, in Iran, the prevalence of HEV infection is considerably low compared to HEV prevalence among pregnant women of the other endemic countries [9].

Our study showed that younger pregnant women were more likely to be anti-HEV IgM seropositive as compared to older women, and the mean age of anti-HEV IgM seropositive pregnant women was significantly lower than that of seronegative women. This is consistent with the age distribution of HEV infection in developing countries, where HEV infection is more prevalent among young adults [33]. The high prevalence of HEV among young population of this territory is a cause for concern. Therefore, improvement of sewage disposal system, public hygiene, sanitation and drinking water supply systems is required to drive down HEV prevalence in the society. Our results in accordance with those of most studies from Iran revealed a significant association between older ages (women over 34 years old in this study) and higher anti-HEV IgG prevalence due to cumulative exposure to the virus over time [8, 15–17, 31]. This finding is also in concordance with several studies from France [29], Turkey [27], Ethiopia [22], Mexico [19] and Egypt [20], which have shown a higher prevalence of HEV among older age groups compared to younger age groups [19, 20, 22, 27, 29].

The results of the present study also demonstrate that uneducated women are more prevalent infected by HEV than educated pregnant women. This decreasing prevalence with education level can be explained by the fact that people with lower education are less aware of HEV infection and the possible ways of exposure to HEV and often live in poor socioeconomic and hygiene conditions, which increase the risk of HEV transmission. The significant association between level of education and HEV seroprevalence in this study is in agreement with the previous studies from Iran [15–17] and the other parts of the world [22, 23, 27, 34]. In contrast, a recent study from Mexico has shown that there is no significant association between level of education and HEV seropositivity [19].

Another risk factor associated with HEV seropositivity is ethnicity. In this study, HEV seroprevalence was more prevalent in Afghan than the other ethnic groups. Afghanistan is considered as a highly endemic country for HEV infection [35]. The high endemicity of HEV infection in Afghanistan also supports the high seroprevalence of HEV infection among Afghan immigrants. Furthermore, we found a significant association between HEV seroprevalence and the number of pregnancy, so that anti-HEV seroprevalence was significantly higher among those women with more than three pregnancies. This finding is consistent with studies done by Mamani *et al* [16] and Tabarraei *et al* [17], while some other studies do not support this finding [14, 22, 25].

Bushehr and Jam showed the highest and the lowest rates of HEV seroprevalence, respectively, as compared to the other cities in this territory. The high prevalence of HEV among pregnant women resident in Bushehr city can be explained by this fact that Bushehr seaport is one of the most important international ports in south of Iran with a high migration flow, while Jam city has the lowest rate of immigration in this territory.

In our study, HEV seropositivity among pregnant women was not statistically associated with history of abortion, stage of gestation, and time of sampling. All seropositive women had successful childbirth, and no maternal, prenatal or neonatal complications were reported. In some geographical regions, HEV infection follows an asymptomatic or mild course in pregnancy. The benign pathogenicity of HEV infection might be due to the predominance of less virulent strains of HEV in these regions [2, 36]. With respect to the fact that genotypic pattern of HEV in Iran is unknown, more studies are required to generalize this conclusion to Iran. In contrast, a recent study from Sudan revealed the significant role of HEV in spontaneous abortion among Sudanese pregnant women [21]. On the other hand, Rasti *et al*, Tabarraei *et al* and Adjei *et al* demonstrated that HEV seropositivity among pregnant women is associated with stage of gestation [15, 17, 23].

The strength of this study is consecutive recruitment of pregnant women, which has increased generalizability of the results to the pregnant population of this territory. As a limitation, we were not able to evaluate the possible association between socioeconomic status of pregnant women and HEV seropositivity, because the majority of pregnant women were not willing to answer this question. Some studies from Turkey and India have reported a significant association between socio-economic status and HEV seropositivity, so that pregnant women with low socioeconomic status tended to be more positive for HEV infection [27, 34]. In contrast, a recent study from Mexico showed that HEV seroprevalence was not associated with socioeconomic status of pregnant women [19]. Furthermore, we could not determine the genotypic pattern of HEV infection among pregnant women. Since none of the pregnant women had HEV viremia, and HEV RNA did not find in any of the samples. HEV viremia is short-lived. In stool, however, the virus can be detected for a longer period of time than in serum [33, 4]. Nevertheless, we did not try to isolate HEV from the stool specimens. This is another limitation of the current study. Therefore, the molecular epidemiology of HEV infection remained unknown in this territory.

## Conclusion

The northern shores of Persian Gulf in Iran, with HEV seroprevalence of 6.3%, can be classified as an endemic geographical region for hepatitis E, and residents of Bushehr city, Afghan immigrants and uneducated women are the main at-risk populations. At present, there are no data on the probable effects of hepatitis E on pregnancy outcomes in terms of maternal and prenatal mortality in Iran. In addition, the circulating HEV genotypes in the pregnant population of Iran need to be determined in order to characterize the epidemiological patterns of HEV in terms of pathogenicity and severity.
